# ITM2A as a potential prognostic marker for triple-negative breast cancer

**DOI:** 10.7150/jca.114801

**Published:** 2025-06-23

**Authors:** Can Jiang, Ruixin Feng, Yongqian Zhao, Jingting Zhang, Na Han, Yuzhu Zhang, Guang Shu, Gang Yin, Maonan Wang

**Affiliations:** 1 Department of Pathology, Xiangya Hospital, Xiangya School of Basic Medical Sciences, Central South University, Changsha, China.; 2 National Clinical Research Center for Geriatric Disorders, Xiangya Hospital, Central South University, Changsha, China.

**Keywords:** triple-negative breast cancer, treatment resistance, ITM2A, Prognostic analysis

## Abstract

Different subtypes of breast cancer pose great challenges for precision therapy, especially triple-negative breast cancer (TNBC), because it lacks effective therapeutic targets and is highly resistant to chemotherapy. In this study, the transmembrane protein ITM2A was systematically identified as a novel prognostic biomarker and potential therapeutic target for TNBC. ITM2A was found to be significantly under expressed in TNBC tissues, as revealed by differential expression profiling. Furthermore, patients exhibiting low ITM2A expression demonstrated worse overall survival (OS), recurrence-free survival (RFS), and distant metastasis-free survival (DMFS). A combined multi-omics analysis revealed a significant association between low ITM2A expression and immunosuppressive microenvironmental features. It is noteworthy that the ITM2A high-expression group exhibited substantial clinical benefits in anti-PD-L1 treatment (AUC=0.982) and CAR-T treatment (AUC=0.827). Gene Ontology functional annotation and KEGG pathway enrichment analysis indicated that ITM2A may coordinate anti-tumor immune responses by regulating copper ion metabolic reprogramming and immune checkpoint networks. Pharmacogenomic analysis further confirmed that the expression level of ITM2A was negatively correlated with the sensitivity of etoposide. By establishing the 'immunometabolism-therapeutic response' regulatory axis of ITM2A, this study hopes to provide an innovative theoretical basis for the targeted treatment of TNBC and the precise stratification of immunotherapy.

## Introduction

With a global incidence of 11.6 percent, breast cancer is one of the most common malignant tumors in women 1. In terms of molecular typing, breast cancer consists of four molecular subtypes: Luminal A, Luminal B, HER2-positive, and triple-negative breast cancer. Among them, the triple-negative breast cancer tends to develop at a younger age, has a high degree of malignancy, is highly invasive, has the worst prognosis of all breast cancers, and has been called "the most toxic breast cancer" 2-5. The intractability of triple-negative breast cancer stems from its complex biology, scarcity of therapeutic targets, and dependence and resistance to conventional chemotherapy 6, 7. Although cutting-edge therapies such as neoadjuvant chemotherapy, PARP inhibitors, and combination immunotherapy have brought hope to some patients in recent years, the treatment of triple-negative breast cancer patients remains ineffective due to key challenges such as high individual heterogeneity, unclear resistance mechanisms and difficulty in accurate typing 8-11. Consequently, the accurate identification of effective targets for triple-negative breast cancer, as well as the development of new targeted therapeutic agents, has become an urgent challenge in triple-negative breast cancer research.

The application of bioinformatics analysis in genomics, disease research, drug development, and other fields is set to become more in-depth and extensive, driven by continuous technological advancement. Bioinformatics analysis provides a powerful tool and platform for the discovery of new targets for disease treatment by integrating multidimensional data and advanced computational methods. In this study, the substantial volume of data in the GEO database was utilized comprehensively. The GEO database is a publicly accessible gene expression data repository that facilitates the sharing and retrieval of high-throughput gene expression, epigenetic, and other functional genomics data. It is extensively employed in the domains of biology, medicine, and pharmacology research 12. Four datasets (GSE45827, GSE65194, GSE42568, GSE38959) were obtained from the GEO database, after which the differentially expressed genes in normal tissues and breast cancer tissues of these four datasets were analyzed. Subsequently, we undertook a prognostic correlation analysis of the differentially expressed genes in triple-negative breast cancer patients. This analysis identified ITM2A, a gene expressed at low levels in breast cancer tissues and associated with prognosis in triple-negative breast cancer patients. ITM2A is a novel type II integral membrane protein that has been implicated in the processes of osteogenic and chondrogenic differentiation 13, 14. It is localized to chromosomal positions Xq13.3-Xq21.2 15. Subsequent investigation was directed towards elucidating the function of ITM2A in influencing the prognosis of patients diagnosed with triple-negative breast cancer. To this end, a comprehensive array of analytical procedures was employed, encompassing enrichment analysis, immune correlation analysis, correlation analysis with major immune checkpoints, and drug sensitivity analysis. The ensuing analyses yielded findings that suggest ITM2A as a promising therapeutic target for triple-negative breast cancer, with potential implications in both immune-related and prognostic-related contexts, as well as in the context of copper-death-related processes. This study is poised to establish novel foundational theories for the treatment of patients with triple-negative breast cancer, a development that is of paramount importance for the design of therapeutic interventions.

## Materials and Methods

### The analysis of differential expression

The UALCAN database (https://ualcan.path.uab.edu/) is a website that has been built on PERL-CGI, JavaScript, and CSS, enabling effective online analysis and mining of cancer data. This software facilitates the execution of comprehensive analyses of gene expression data from cancer patients, with a primary focus on the TCGA database of pertinent cancer data. It assists medical professionals in the identification of biomarkers, the analysis of gene expression, survival analysis, and related tasks. Additionally, it enables the retrieval of relevant information from other databases via associated links. We used this database to analyze the expression levels of ITM2A in breast cancer tissues compared to normal tissues, as well as the expression levels of ITM2A in different molecular types of breast cancer.

### Prognostic analysis

The KM Plotter database (https://kmplot.com/analysis/) is an online platform designed for biomedical research, primarily for analyzing the relationship between gene expression and survival prognosis of tumor patients. The database under consideration integrates a substantial amount of public gene expression and clinical data (TCGA, GEO, etc.). It employs the Kaplan-Meier survival analysis method to assess the correlation between gene expression levels and clinical indicators, such as overall survival and progression-free survival of patients. This method is advantageous as it facilitates the validation of the prognostic relevance of biological markers for candidate genes. The present study utilized a substantial database to conduct an analysis of the impact of ITM2A on three key metrics: overall survival (OS), recurrence-free survival (RFS), and distant metastasis-free survival (DMFS). These metrics were examined in patients diagnosed with triple-negative breast cancer.

### Enrichment analysis

The STRING database (https://cn.string-db.org/) is a comprehensive public database focusing on protein-protein interactions (PPIs). It has been designed to assist researchers in predicting and analyzing functional associations and physical interactions between proteins by integrating multiple data sources. The functions of proteins whose sequence is not yet known can be predicted, and potential roles can be inferred from the known functions of the proteins with which they interact. The database under consideration here explores genes that interact with ITM2A, and from which network diagrams of interactions with ITM2A are obtained.

The DAVID database (https://davidbioinformatics.nih.gov/) is an online bioinformatics research tool that provides comprehensive gene function annotation information, integrating the annotation contents of several authoritative databases, including the Gene Ontology, KEGG Pathway, BioCarta Pathway, and others. It facilitates rapid acquisition of detailed descriptions of genes in terms of biological processes, molecular functions, cellular components, and so on. Utilizing the DAVID database's advanced annotation and analysis capabilities, we have employed the database to functionally annotate the genes interacting with ITM2A, with the objective of elucidating the function of ITM2A.

BioLadderv2.0 Bioinformatics Online Analysis Cloud Platform (https://www.bioladder.cn/v2/#/firstVue) is a comprehensive bioinformatics analysis tool that provides users with convenient data processing, functional analysis, and visualization. It covers more than 30 chart types to meet basic and advanced visualization needs. The platform was used to visualize the enrichment results analyzed.

### The analysis of correlation in the field of immunology

The BEST database (https://rookieutopia.hiplot.com.cn/app_direct/BEST/) is a tool designed for the analysis of the stability of biomarkers in tumors. It comprises eight analysis modules, encompassing 27 tumors and 363 cohorts. The database allows for the entry of individual genes or gene collections, alongside the provision of clinically relevant data. This functionality enables the exploration of the clinical significance and biological functions of cancer biomarkers in a comprehensive and systematic manner. In this study, the database was utilized to conduct an in-depth analysis of the ITM2A gene, exploring its correlation with immune cell infiltration, sensitivity to immunotherapy, and related biological processes.

The TIMER database (http://timer.comp-genomics.org/) is a comprehensive online analytical platform that provides Immune Association, Cancer Exploration, and Immune Estimation modules. The purpose of these modules is to investigate associations between immune infiltration and genetic or clinical characteristics. The database is based on powerful analytical capabilities, and the present study used it to analyze the correlation between ITM2A and immune cell infiltration in triple-negative breast cancer.

### Drug sensitivity analysis

The GSCA database (https://guolab.wchscu.cn/GSCA/) is a comprehensive bioinformatics analysis platform focusing on cancer research. It integrates multi-omics data (e.g. gene expression, mutation, immune infiltration, drug susceptibility) and provides visualization tools to help researchers rapidly mine biomarkers. The database's features, including its comprehensiveness and efficiency in integrating various omics data, have been utilized to analyze the drug sensitivity of ITM2A.

### Molecular docking

The PubChem database (https://pubchem.ncbi.nlm.nih.gov/) is a freely available chemical database that is maintained by the National Center for Biotechnology Information (NCBI). It currently contains over 111 million compounds, 294 million substance entries, and millions of biological experimental datasets. The present study used this database to obtain the molecular structure of etoposide.

Schrödinger Maestro is a full-featured software package that supports drug design, molecular dynamics simulation, virtual screening, and other studies, and is widely used in drug discovery, materials science, and biomolecular simulation. This software was used to achieve molecular docking of etoposide with the ITM2A.

### Cell culture

The triple-negative breast cancer cell line (HCC1086) used in the experiments was obtained from the American Type Culture Collection (ATCC). the HCC1086 cell line was cultured in RPMI 1640 medium containing 10% fetal bovine serum and 1% penicillin/streptomycin, all in a humidified incubator at 37 degrees Celsius with 5% CO2. All cells were free from mycoplasma, bacterial and fungal contamination.

### CCK8

The plasmids were synthesized by Miaoling Biosynthesis (Wuhan, China) and then introduced into the HCC1806 cell line using the HiPerFect kit (QIAGEN GmbH, Netherlands), in accordance with the manufacturer's instructions. Following a 24-hour transfection period, the cells were seeded into 96-well plates at a cell volume of 1 × 10^4 per well. Thereafter, the drug etoposide was added according to the gradient after the cells were attached to the wall. Forty-eight hours later, CCK8 was measured, and the IC_50_ was calculated.

### Statistical analysis

Data were analyzed using GraphPad Prism 10 software. p<0.05 was set as the criterion for significance.

## Results and Discussion

### Genetic screening

**Figure 1:**
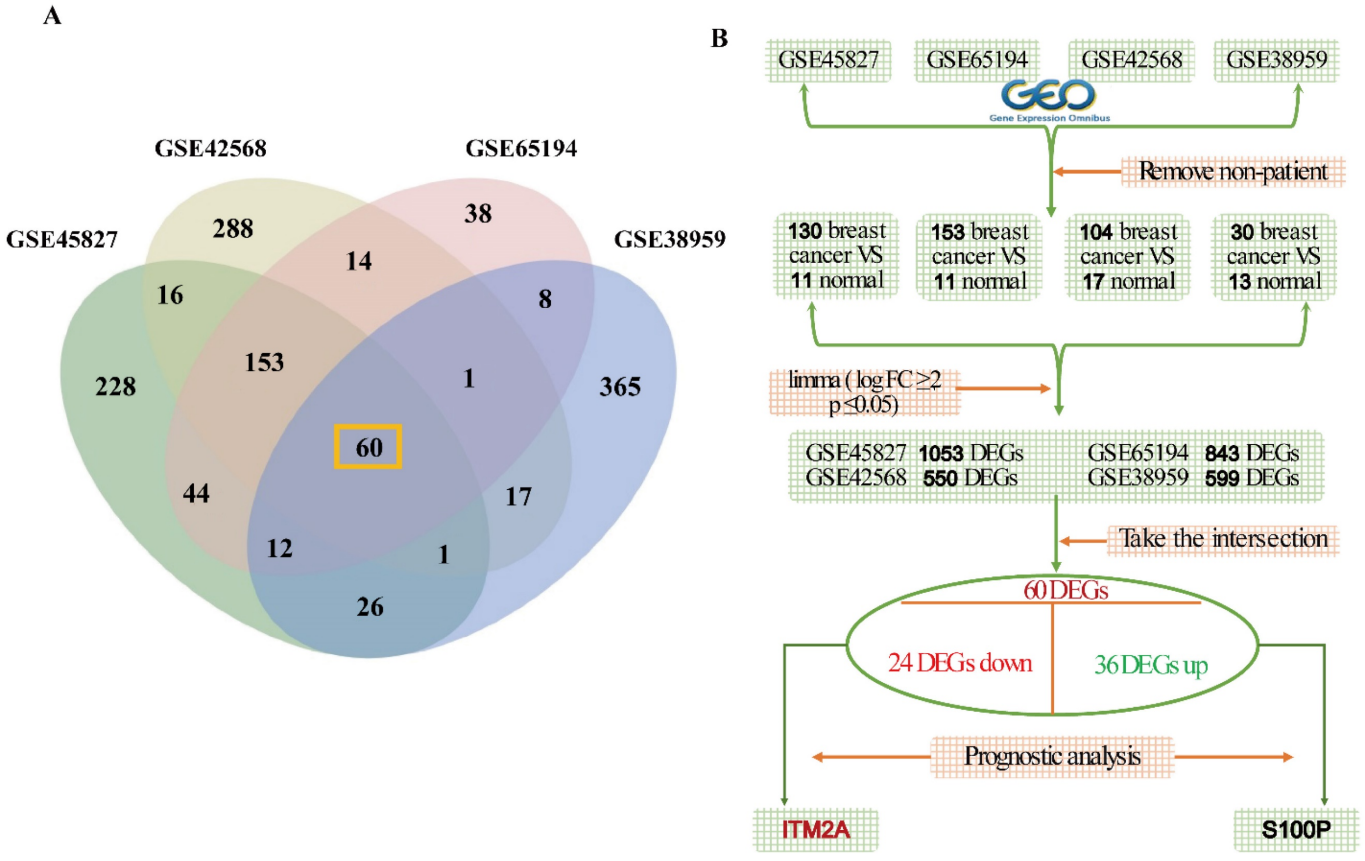
Schematic diagram of the gene screening process. **(A)** Venn diagram of gene intersection analysis based on four independent data sets; **(B)** multi-step screening process to finalize the ITM2A.

Four datasets were retrieved from the GEO database: GSE45827 (consisting of 130 breast cancer samples and 11 normal samples), GSE65194 (consisting of 153 breast cancer samples and 11 normal samples), GSE42568 (consisting of 104 breast cancer samples and 17 normal samples), GSE38959 (consisting of 30 breast cancer samples and 13 normal samples), and the specific flowcharts for the analysis of variance are mentioned (Figures 1A-1B). The following detailed steps were taken in the course of the study: firstly, the limma R package was utilized in order to analyze the differentially expressed genes between breast cancer tissues and normal tissues, with the criteria for this analysis being |logFC|≥2 and P≤0.05. The results of the screening process revealed 1053 differentially expressed genes (DEGs) in dataset GSE45827, 843 DEGs in dataset GSE65194, 550 DEGs in dataset GSE42568, and 599 DEGs in dataset GSE38959. To explore the DEGs with general significance in breast cancer, the intersection of these four datasets was determined, resulting in the identification of a total of 60 DEGs (Figure 1A). After this determination, the 60 differentially expressed genes were grouped according to their high and low expression in breast cancer patients, yielding a total of 36 genes with up-regulated expression and 24 genes with down-regulated expression in breast cancer. In addition, we conducted prognostic analyses of the 60 DEGs in these two groups. Ultimately, we identified one clinically significant prognostic biomarker in each subgroup: an upregulated gene (S100P) associated with poor survival and a downregulated gene (ITM2A) correlated with favorable outcomes in breast cancer patients.

A review of the extant literature revealed that S100P expression has been documented in numerous types of cancer, and its expression has been linked to drug resistance, metastasis, and poor clinical outcomes 16. A body of research has demonstrated that elevated expression of this specific gene is associated with a poor prognosis and functions as a prognostic indicator for triple-negative breast cancers 17. Furthermore, studies have demonstrated that S100P, in conjunction with Ezrin, influences the migration of triple-negative breast cancer cells across the endothelium 18. ITM2A, also known as Integral Membrane Protein 2A, is a protein-coding gene that encodes a type II membrane protein belonging to the ITM2 family. Mouse-related studies have demonstrated that this gene may play a role in osteogenic and chondrogenic differentiation 13, 14. To date, there has been no significant advancement in the field of ITM2A research related to triple-negative breast cancer. To discover potential therapeutic targets for improving clinical outcomes in TNBC, this study has focused on ITM2A as a novel candidate, with mechanistic exploration of its prognostic and druggable properties.

### ITM2A expression is low in triple-negative breast cancer, and it positively correlates with the patient's poor prognosis

**Figure 2:**
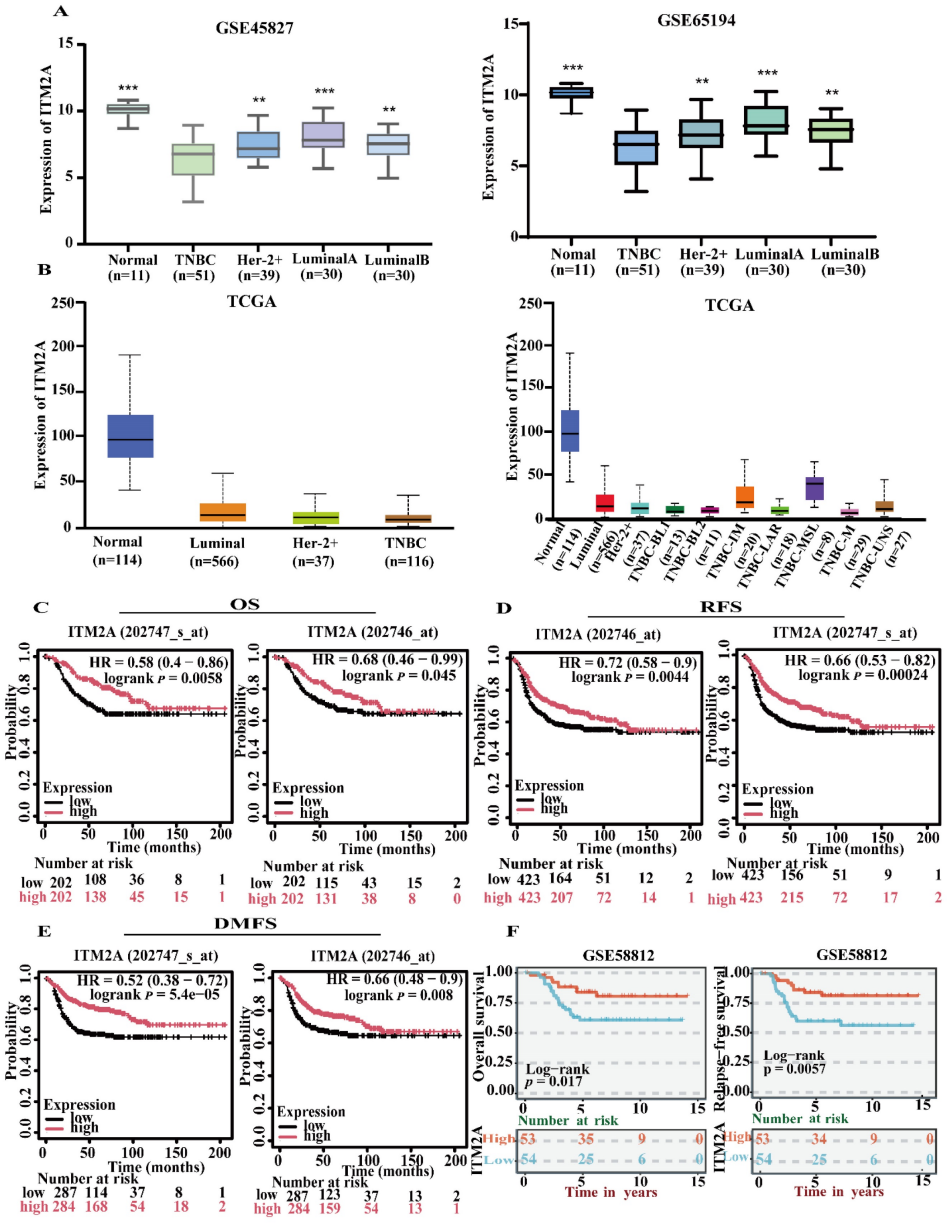
Low expression of ITM2A in triple-negative breast cancer and positive correlation with poor patient prognosis. (A) GEO database (GSE45827, GSE65194) in different breast cancer subtypes, analysis of expression level of ITM2A (*p < 0.05, **p < 0.01, ***p < 0.001); (B) TCGA database, analysis of differential expression of ITM2A in different breast cancer subtypes (p<0.05); (C-E) KM-based Plotter database for survival analysis: positive correlation of ITM2A mRNA expression level with overall survival, recurrence-free survival and distant metastasis-free survival in TNBC patients; (F) BEST database to validate the prognostic associations of ITM2A mRNA expression with OS and RFS in TNBC patients.

In this study, we initiated our research by conducting a re-analysis of the ITM2A expression data from the GEO database. This analysis revealed that the expression level of ITM2A was significantly lower than that of other subtypes of breast cancer (Figure 2A); Furthermore, we sought to validate the expression levels of ITM2A in normal tissues versus breast cancer tissues and other molecular subtypes of their breast cancers using the online database UALCAN (https://ualcan.path.uab.edu/). This analysis revealed that the expression levels of ITM2A in the TCGA database were significantly lower than those in normal breast tissues or other breast cancer molecular subtypes (*p* < 0.05) (Figure 2B). Up to this point, we have validated the low expression of ITM2A in a total of 136 normal samples and 218 triple-negative breast cancer samples.

In the following study, the hypothesis was investigated of whether the expression level of ITM2A has an effect on the prognosis of patients diagnosed with triple-negative breast cancer. Utilizing Kaplan-Meier analysis, we ascertained that patients with triple-negative breast cancer who exhibited low ITM2A expression demonstrated inferior overall survival (OS), recurrence-free survival (RFS), and distant metastasis-free survival (DMSF) in comparison to those with high ITM2A expression (Figure 2C-2E). A subsequent analysis of the data from the GEO database yielded similar results, indicating that patients with triple-negative breast cancer exhibited a relatively poor prognosis when ITM2A expression level was low (Figure 2F). This finding is consistent with the established role of ITM2A in bladder cancer and ovarian cancer tissues, where its low expression is associated with poor patient prognosis 19-21. In summary, the collective findings underscore the notion that ITM2A expression level, when diminished, serve as a prognostic indicator for triple-negative breast cancer, potentially serving as a biomarker for clinical decision-making.

### GO annotation and KEGG pathway enrichment analysis of ITM2A

**Figure 3:**
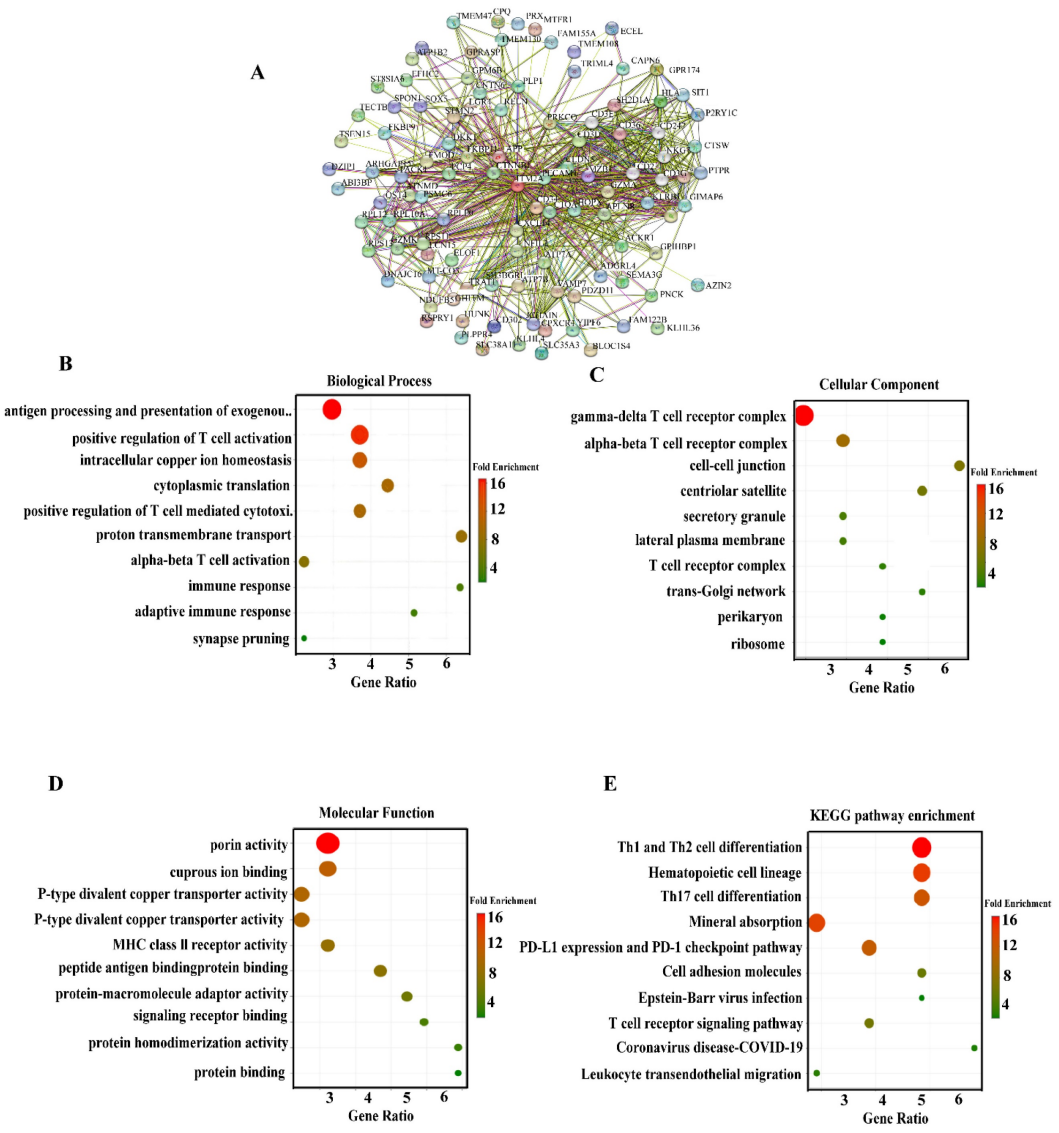
ITM2A protein interaction network, GO functional annotation and KEGG pathway enrichment in triple-negative breast cancer. **(A)** Construction of the ITM2A core protein interaction network (PPI); (B-D) GO functional annotations: biological process **(B)**, cellular component **(C)**, and molecular function **(D)** Visualization of the enrichment results of the top 10 entries; **(E)** Bubble diagram of the top 10 significant pathways for KEGG pathway enrichment analysis. Graphical parameters: Bubble size corresponds to the number of enriched genes, and color depth reflects the degree of enrichment.

Enrichment analysis is a method of determining whether a target gene set is significantly enriched in a particular biological function by comparing it to a background gene set 22. This process can translate gene-level data into functional-level biological explanations, thereby facilitating a more comprehensive understanding of the potential roles of gene sets in cellular activities, disease mechanisms, or molecular functions. The exploration of the interaction proteins of specific genes is not only the core method to understand the function of the genes, but also the bridge connecting the molecular mechanism and phenotype, disease, and treatment. This can better clarify the pivotal position of the genes in the regulatory network and reveal the pathogenic mechanism of the disease-related genes. To further explore the functional role of ITM2A in triple-negative breast cancer, we enriched and analyzed the reciprocal proteins of this gene. The STRING database (https://cn.string-db.org/) was initially utilized to investigate the genes that interact with ITM2A, resulting in the formulation of a protein-protein interaction (PPI) network diagram (Figure 3A). Furthermore, we conducted an enrichment analysis of the top 100 genes that interact with ITM2A using the DAVID database (https://david.ncifcrf.gov/) and subsequently rendered the top 10 enrichment results as a visual display. The results of the biological process enrichment analysis indicated that the corresponding genes were predominantly associated with antigen-antibody processing, intracellular copper ion homeostasis, positive regulation of immune cell activation, and immune response processes (Figure 3B); The cellular component enrichment results demonstrated that the interacting genes of ITM2A were predominantly associated with various immune cell complexes, secretory granules, cell membranes, and ribosomes (Figure 3C); The molecular function enrichment results demonstrated that its interacting genes were frequently implicated in binding to copper ions, associated with bivalent copper transporter protein activity, with MHC class I receptor activity, and with signaling receptor binding (Figure. 3D); A comprehensive analysis of the KEGG database revealed that the interacting genes of ITM2A were predominantly associated with differentiation, mineral uptake, PD-L1 expression, and the PD-L1 check pathway, as well as the T-cell receptor signaling pathway in Th1, Th2, and Th17 cells (Figure 3E).

The present study will focus on the proteins that interact with ITM2A, including the CD family (CD247 23, CD74 24, CD3D 25, etc.) and the HLA family (HLA-F, HLA-C, HLA-DRA, HLA-DOA 26, etc.), and the chemokine receptor CXCR14 27. Previous studies have demonstrated that many of these genes are implicated in the body's immune response and tumor immune escape, among other functions, and are considered classical immune-related molecules. It is noteworthy that the enrichment results indicated the involvement of ITM2A intercalating proteins in copper ion transport and intracellular copper ion homeostasis. The focus was directed towards copper-transporting P-type adenosine triphosphatases (ATP7A, ATP7 B), which are recognized as copper ion transport proteins 28, 29. Therefore, we hypothesize that there may be a relevant mechanism of action between ITM2A and copper ion metabolism in patients with triple-negative breast cancer. In addition, it has been reported that copper ions can trigger the lysosome-dependent autophagy pathway 30. Consequently, further exploration of the mechanisms associated with ITM2A and its copper ion metabolism is anticipated to furnish a theoretical foundation of considerable pertinence for the management of triple-negative breast cancer patients. The collective findings indicate that ITM2A may influence the progression and drug sensitivity of triple-negative breast cancer patients by modulating tumor immunity and body metabolism. This suggests that further elucidation of the mechanisms related to tumor immunity and body metabolism of ITM2A may render ITM2A a potential therapeutic target for triple-negative breast cancer.

### Correlation analysis of immune cell infiltration and classical immune checkpoints with ITM2A mRNA expression levels

**Figure 4:**
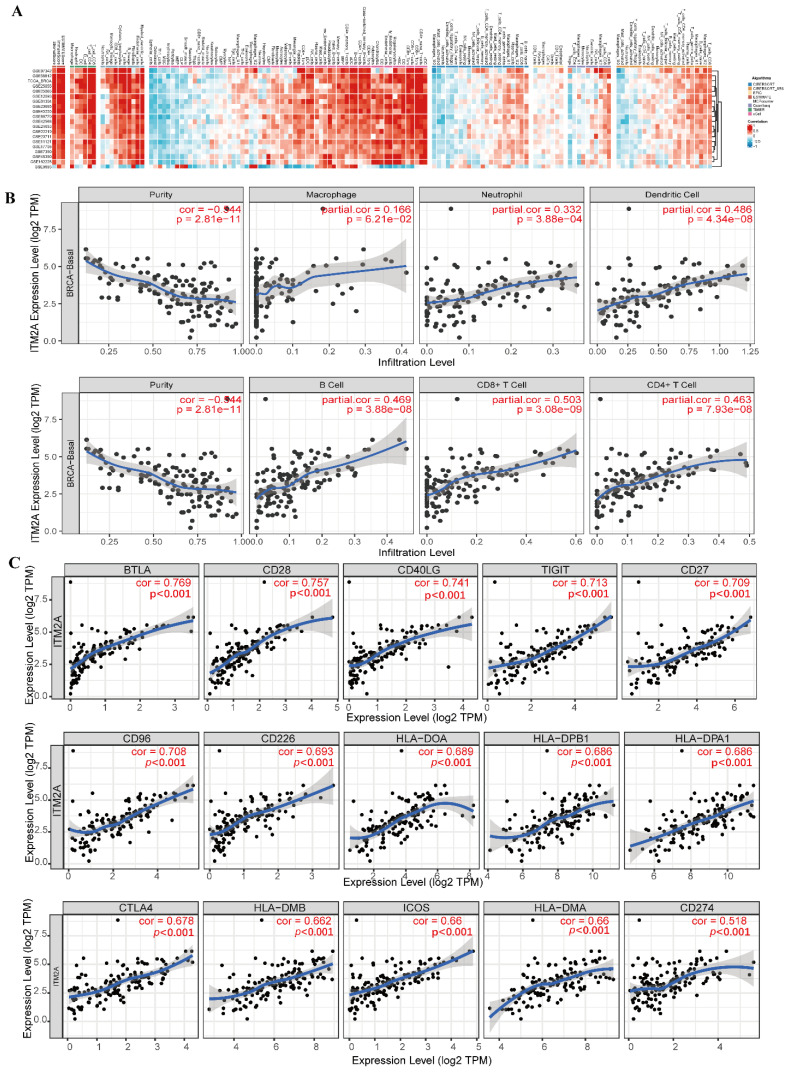
Multi-database joint analysis of ITM2A and immune microenvironment characterization in triple-negative breast cancer. **(A)** Multi-algorithm consistency assessment of immune infiltration based on the BEST database: eight computational methods show the heatmap of the correlation between ITM2A and tumor-infiltrating immune cells (color gradient indicates Spearman's correlation coefficients); **(B)** Analysis of the correlation between ITM2A and immune cells in triple-negative breast cancer in the TIMER database. (B cells (Cor=0.469,* p*<0.001), CD8+ T cells (Cor=0.503, *p*<0.001), CD4+ T cells (Cor=0.463, *p*<0.001), macrophages (Cor=0.166, *p*<0.001), neutrophils (Cor=0.332, *p*<0.001), dendritic cells (Cor=0.486, *p*<0.001)) **(C)** Top 15 molecular correlation analysis of ITM2A and immune checkpoints.

The preceding results indicate a potential association between ITM2A and tumor immunity. To further investigate the relationship between ITM2A and cellular immunity, an analysis was conducted of the relationship between the expression level of ITM2A and the infiltration of immune cells in the BEST database (https://rookieutopia.hiplot.com.cn/app_direct/BEST/). Different datasets were presented in the form of heat maps of immune cell infiltration (Figure 4A). An analysis of the BEST database was conducted using eight distinct algorithms to assess the level of immune cell infiltration. The analysis revealed a positive correlation between ITM2A and most immune cell infiltrations.

Subsequently, we further explored the expression level of ITM2A with the infiltration level of immune cells in the TIMER database (http://timer.comp-genomics.org/). We found that in triple-negative breast cancers, the expression level of ITM2A was associated with the infiltration level of B-cells (Cor=0.469, *p*<0.001), CD8+ T-cells (Cor=0.503, *p*<0.001), CD4+ T cells (Cor=0.463, *p*<0.001), macrophages (Cor=0.166, *p*<0.001), neutrophils (Cor=0.332, *p*<0.001), and dendritic cells (Cor=0.486, *p*<0.001). The TIMER database further revealed a positive correlation between ITM2A expression levels and the infiltration levels of immune cells in breast cancer (Figure 4B). These findings suggest that the expression level of ITM2A may influence the infiltration of immune cells in the immune microenvironment of triple-negative breast cancer.

Subsequently, a review of the literature yielded the identification of classical immune checkpoint molecules 31. An analysis was then conducted to ascertain the correlation between the expression levels of ITM2A and those of classical immune checkpoint molecules. This analysis was conducted using the TIMER database (http://timer.comp-genomics.org/). The results demonstrated a positive correlation between the expression levels of ITM2A and more than 60 classical immune checkpoint molecules, including PD-L1 (CD274), CTLA4, and LAG3 (Table 1) (Figure 4C). PD-L1 (CD274), CTLA4, and LAG3 are all targeted drugs that are used in clinical treatments. Tumor progression is the result of its dynamic interaction with the immune system 32. In this interaction, a complex immune escape mechanism can be developed to evade the killing of tumor cells by the immune system 33.

Despite the demonstrated efficacy of immunotherapy in enhancing the body's anti-tumor immune function and its application in a wide range of tumors, the challenges of low efficacy and a limited patient population persist. Consequently, there is a necessity to explore additional biotherapeutic targets to enhance the efficacy of treatment 34, 35. Research has demonstrated that the expression of ITM2A in mouse thymocytes leads to a reduction in CD8 expression in CD4+CD8+ double-positive thymocytes 36. The extant results indicate a positive correlation between ITM2A mRNA expression levels and immune cell infiltration in triple-negative breast cancers, as well as with more than 60 classical immune checkpoint molecules, including PD-L1 (also known as CD274). Immune checkpoint molecules play a key role in the process of immune response, influencing its initiation, regulation, and termination. Tumor cells utilize immune checkpoint molecules to impede the functionality of immune cells, thereby facilitating immune evasion. Clinical therapy aims to restore the anti-tumor function of immune cells by intervening in the binding of immune checkpoint molecules and their ligands, consequently enhancing the survival rate of tumor patients 37-40. Zhang R et al. reported that ITM2A influenced PD-L1 expression in breast cancer 41. Therefore, it is reasonable to hypothesize that ITM2A may influence tumor progression by affecting the infiltration level of immune cells and the expression of related immune checkpoint molecules in triple-negative breast cancer. Overall, it is of great significance to further explore the tumor immune mechanism of ITM2A in triple-negative breast cancer to make ITM2A a potential immune-related therapeutic target in triple-negative breast cancer and to improve the prognosis of triple-negative breast cancer patients.

### Analysis of ITM2A expression level with immunotherapy sensitivity and prognostic value

**Figure 5:**
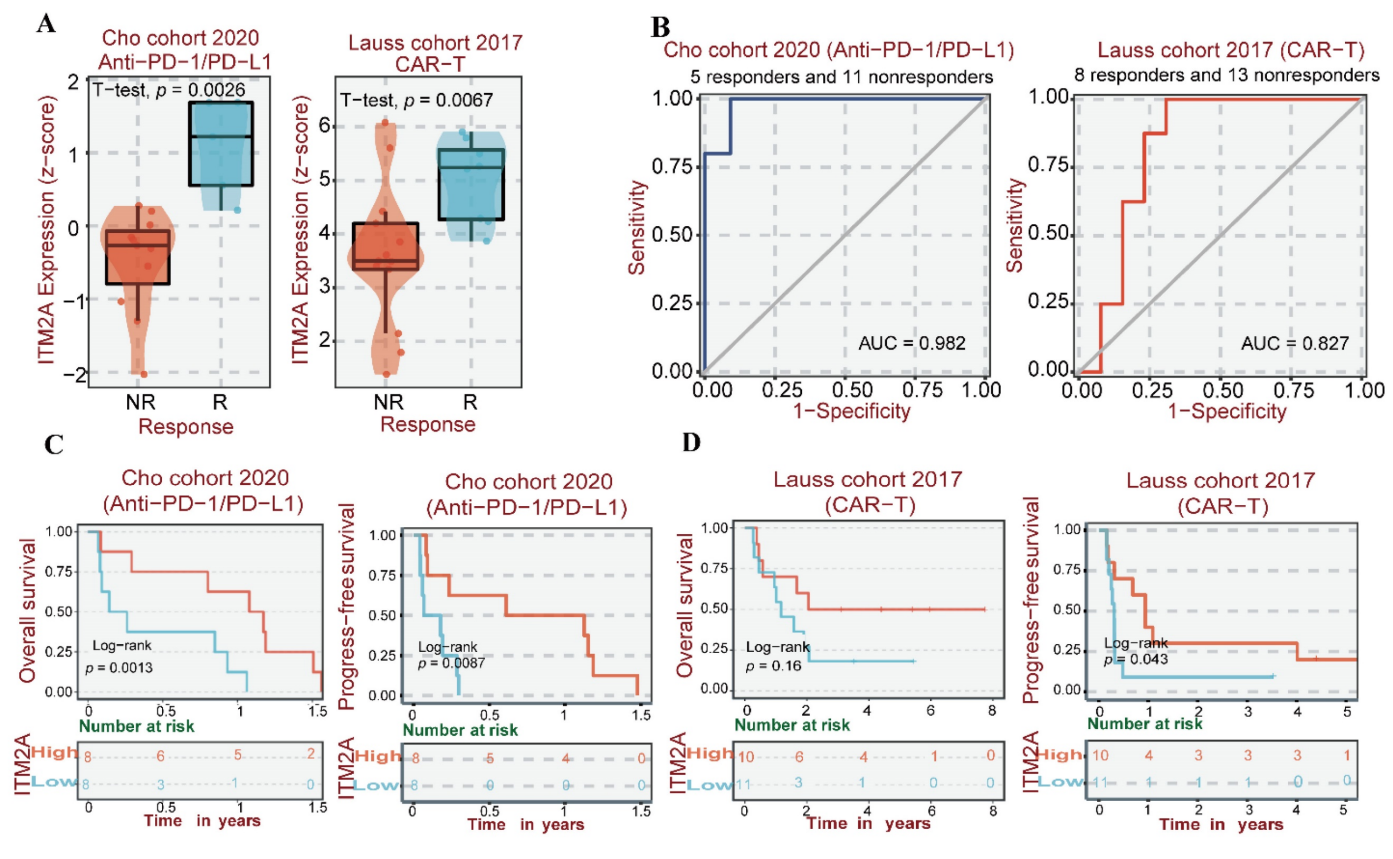
** (A)** Multi-cohort immunotherapy response correlation analysis: difference in treatment response between ITM2A expression levels in Cho cohort (anti-PD-L1 therapy) and Lauss cohort (CAR-T therapy); **(B)** ROC curve analysis of ITM2A predicting clinical benefit of immunotherapy (AUC=0.82, 95% CI 0.76-0.88), where the horizontal axis is the false positive rate (specificity) and the vertical axis is the true positive rate (sensitivity); **(C)** Survival curves of ITM2A expression versus anti-PD-L1 treated patients in the Cho cohort, where red indicates high expression and blue indicates low expression. **(D)** Survival curves of ITM2A expression versus CAR-T therapy patients in the Lauss cohort, red indicates high expression and blue indicates low expression. (Log-rank *p* < 0.05)

Tumor immunotherapy constitutes an innovative therapeutic approach that consists of immune checkpoint inhibitors (ICIs) and chimeric antigen receptor (CAR) T-cell therapy 42-44. ICIs are primarily anti-PD-L1 therapies and CAR-T cell therapies, which have been used in part to treat triple-negative breast cancers 45, 46. Therefore, given the hypothesis that ITM2A may be associated with the immune microenvironment of patients with triple-negative breast cancer, we conducted a further analysis of the expression levels of ITM2A in patients who exhibited a response to anti-PD-L1 therapy and those who did not respond to CAR-T cell therapy. By examining data from the Cho cohort in the BEST database, we analyzed and found that responding patients to anti-PD-L1 therapy in this cohort had higher ITM2A expression levels compared to non-responding patients (*p*<0.05). When analyzing the Lauss cohort, we found that patients in this cohort who responded to CAR-T therapy also had higher ITM2A expression levels compared to non-responders (*p*<0.05) (Figure 5A). We then analyzed the relationship between immunotherapy and ITM2A expression levels and found that patients with higher ITM2A expression levels in this cohort had significantly better OS and RFS after anti-PD-L1 therapy and CAR-T therapy (Figure 5C-D). In addition, the Receiver Operating Characteristic (ROC) curves for both cohorts reflect the ability of ITM2A to discriminate between anti-PD-L1 therapy and CAR-T therapy responders and non-responders, with the area under the curve (AUC=0. 982) for the Cho cohort (anti-PD-L1 therapy) and the area under the curve (AUC=0.827) for the Lauss cohort (CAR-T therapy) reflect the fact that ITM2A may be a good target for identifying triple-negative breast cancer patients who respond to immunotherapy (Figure. 5B). The high expression of ITM2A in triple-negative breast cancer is associated with a high infiltration of immune cells and a high sensitivity to relevant immunotherapy. These findings suggest that ITM2A has the potential to serve as an effective therapeutic target for triple-negative breast cancer.

### Drug sensitivity analysis of ITM2A and molecular docking

**Figure 6:**
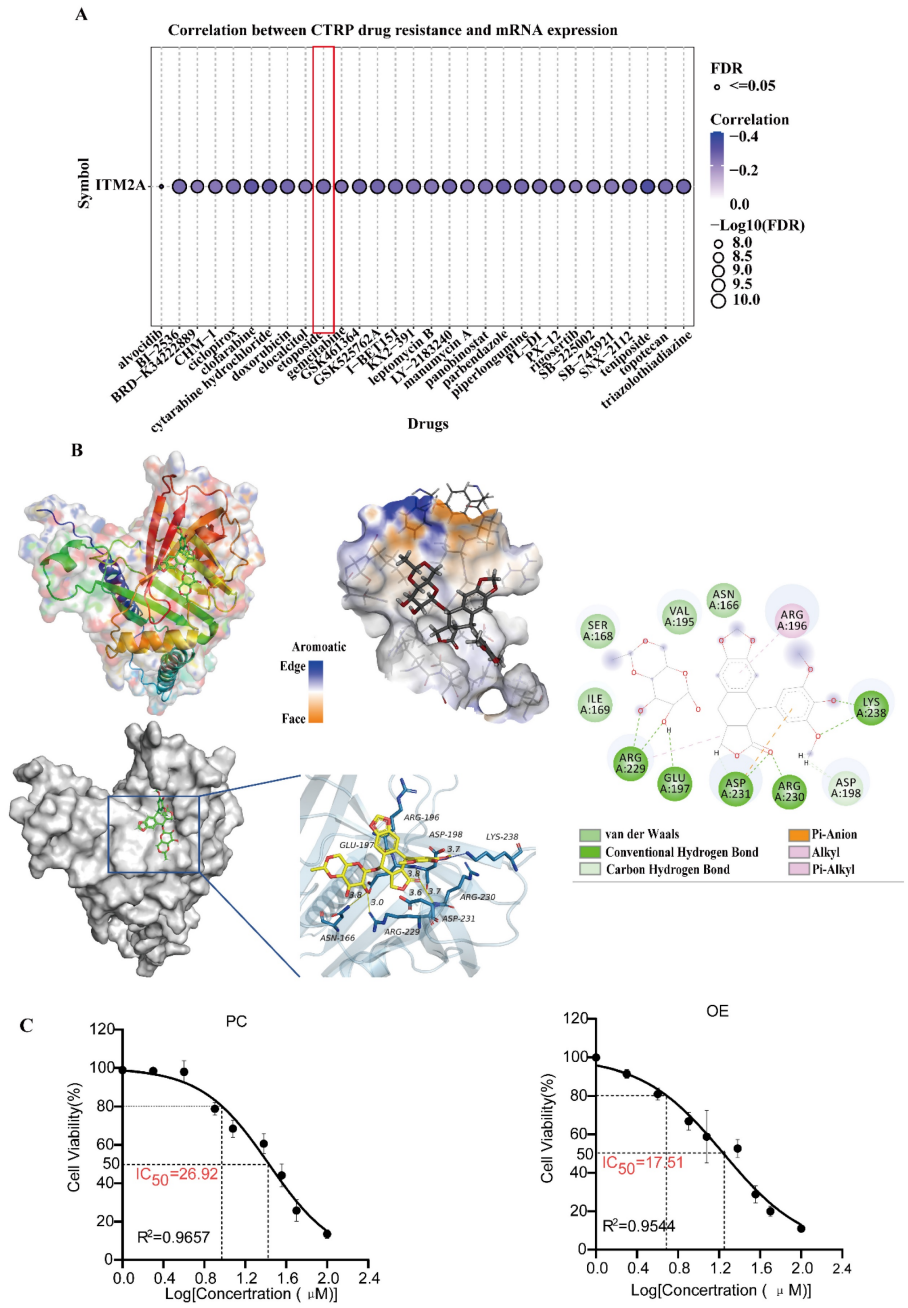
Validation of the pharmacological characterization and targeted therapeutic potential of ITM2A (**A**) ITM2A-associated drug sensitivity prediction based on the CTRP database, AUC > 0.2 and FDR < 0.05; (**B**) Validation of the molecular docking binding mode of the ITM2A protein to etoposide, binding energy ΔG = -6.76 kcal/mol. Important hydrogen bonding interactions are indicated by dashed lines. (**C**) Determination of IC_50_ values of etoposide added to HCC1806 cells (PC: IC_50_=26.92μM, R^2^=0.9657; OE: IC_50_=17.51, R^2^=0.9544).

Given the high degree of drug resistance exhibited by patients with triple-negative breast cancer, the efficacy of numerous drug therapies is rendered ineffective. Addressing this issue would result in a substantial improvement in the prognosis of patients diagnosed with triple-negative breast cancer. Prior studies have demonstrated that high expression of ITM2A in cervical cancer enhances the sensitivity of patients to cisplatin 47 and that low expression of ITM2A in chronic granulocytic leukemia may be a crucial factor contributing to patients' resistance to imatinib 48. Therefore, the question arises as to whether the low expression of ITM2A in triple-negative breast cancer affects the sensitivity of therapeutic agents utilized in patients with this condition. An analysis was conducted to assess the sensitivity of ITM2A to CTRP drugs. The GSCA database (https://guolab.wchscu.cn/GSCA/) was utilized for this analysis. The results demonstrated a negative correlation between the expression level of ITM2A and the resistance to the single-agent chemotherapeutic drug etoposide in triple-negative breast cancer. That is to say, an increase in the expression level of ITM2A enhanced the drug sensitivity of etoposide in patients with triple-negative breast cancer (Figure 6A). Etoposide, a cell cycle-specific antitumor drug that functions by forming complexes with topoisomerases to impede DNA repair 49, is the prevailing chemotherapeutic agent employed in the treatment of triple-negative breast cancer 50.

In order to investigate the binding effect of etoposide to ITM2A, molecular docking of the drug etoposide with the ITM2A protein was performed. The results of the molecular docking study demonstrated a strong binding effect and a favorable match between etoposide and the target protein, with a binding energy of -6.76 kcal/mol. The complex formed by the compound and protein after docking was visualized using Pymol 2.1 software to obtain the binding pattern of the compound and protein. Based on the binding pattern, the amino acid residues of the compound combined with the protein pocket can be clearly seen. Etoposide compounds have been shown to form robust hydrogen-bonding interactions with the active site amino acids of ITM2A protein (LYS-238, ARG-230, ASP-231, GLU-197, ARG-229), thereby effectively anchoring small molecules within the protein pocket; It is noteworthy that etoposide is also able to form hydrophobic interactions with ARG-196, ARG-229, and in particular, the benzene ring of etoposide forms a pi-anion conjugation with ASP-231, which is important for stabilizing small molecules (Figure 6B). In addition, the HCC1806 cell line was examined, and the IC_50_ of etoposide was ascertained. The results demonstrated that an elevated expression level of ITM2A could indeed enhance the drug sensitivity of breast cancer cells to etoposide (Figure 6C). The aforementioned results suggest that ITM2A may have direct binding with etoposide, thereby degrading the drug and reducing its therapeutic sensitivity. Resolving etoposide resistance would significantly improve the prognosis of chemotherapy for patients with triple-negative breast cancer.

## Conclusions

In this study, we sought to contribute to the resolution of critical issues in the clinical management of triple-negative breast cancer, including the dearth of molecularly targeted therapeutic agents, chemotherapy resistance, and poor prognosis. We employed a systematic approach to elucidate the dual regulatory mechanism of the transmembrane protein ITM2A as a novel therapeutic target for TNBC by integrating a multidimensional bioinformatics analysis system. A comprehensive analysis of data from the TCGA, GEO, and other multicenter cohorts has revealed that ITM2A exhibits distinct low expression patterns in TNBC tissues, which is notably lower compared with other breast cancer subtypes (*p*<0.001).

Furthermore, the low expression status of ITM2A has been found to be significantly and positively correlated with patients reduced overall survival, recurrence-free survival, and distant metastasis-free survival. A multidimensional analysis revealed that ITM2A may affect the progression of TNBC through two pathways: 1) an immune regulation axis, which involves the mediation of immune cell infiltration (including CD8+ T cells, CD4+ T cells, B cells, etc.), the influence of the expression of immune checkpoint molecules (e.g., PD-L1), and the significant elevation of the response rate of immune checkpoint inhibitors; and 2) a metabolic regulation axis, which is involved in the regulation of copper ion homeostasis. Pharmacogenomic analysis further revealed a dose-dependent correlation between the expression level of ITM2A and the sensitivity of etoposide. The novelty of this study lies in the construction of the theoretical framework of "immune-metabolic" two-dimensional targeted therapy. This framework provides prognostic and targeted biomarkers for TNBC and lays a theoretical foundation for the development of combined therapeutic strategies based on the ITM2A regulatory network.

## Figures and Tables

**Table 1:** Assessment of ITM2A expression correlation with immune checkpoint molecules

Cell	Gene Symbol	Cor	*P*	Gene Symbol	Cor	*P*
T cell	CD28	0.757	<0.001	PDCD1	0.603	<0.001
	CD40LG	0.741	<0.001	TNFSF4	0.529	<0.001
	CD27	0.709	<0.001	TNFRSF14	0.502	<0.001
	CD96	0.708	<0.001	ADORA2A	0.450	<0.001
	CD226	0.693	<0.001	TNFSF18	0.420	<0.001
	CTLA4	0.678	<0.001	TNFRSF4	0.367	<0.001
	ICOS	0.660	<0.001	TNFRSF18	0.189	0.026
	TNFSF14	0.658	<0.001	TNFSF9	0.152	0.073
	C10ORF54	0.655	<0.001	ICOSLG	0.067	0.428
	TNFRSF9	0.612	<0.001			
Depletion of T cells	TIGIT	0.713	<0.001	LAG3	0.545	<0.001
	PDCD1	0.603	<0.001	HAVCR2	0.626	<0.001
γδT cell	BTLA	0.769	<0.001	BTNL9	0.469	<0.001
	BTN3A1	0.561	<0.001	BTN2A1	0.220	0.009
	BTN2A2	0.476	<0.001	BTNL3	0.215	0.011
B cell	CD86	0.609	<0.001	CD209	0.518	<0.001
	CD40	0.578	<0.001	CD70	0.240	0.004
	CD80	0.553	<0.001			
NK cell	CD226	0.693	<0.001	KIR2DL4	0.425	<0.001
	KIR3DL1	0.563	<0.001	KIR2DL1	0.354	<0.001
	CD160	0.524	<0.001	KIR2DS4	0.300	<0.001
	KIR3DL2	0.495	<0.001	KIR3DL3	0.253	0.003
	KIR2DL3	0.454	<0.001	CEACAM1	0.048	0.574
Dendritic cell	HLA-DOA	0.689	<0.001	HLA-DQB1	0.619	<0.001
	HLA-DPB1	0.686	<0.001	HLA-DQA1	0.613	<0.001
	HLA-DPA1	0.686	<0.001	HLA-DRB1	0.598	<0.001
	HLA-DMB	0.662	<0.001	HLA-DRB5	0.537	<0.001
	HLA-DMA	0.660	<0.001	HLA-DOB	0.469	<0.001
	HLA-DRA	0.655	<0.001	CD276	-0.112	0.189
Macrophage	PDCD1LG2	0.607	<0.001	CD47	0.286	<0.001
	LGALS9	0.524	<0.001	SIRPA	0.232	0.006
	IDO1	0.540	<0.001	VTCN1	-0.068	0.424
Cancer cell	CD274	0.518	<0.001	CEACAM1	0.048	0.574
	TDO2	0.452	<0.001	PVR	-0.113	0.184

Note: This is a Spearman correlation analysis based on the TIMER database.

## Abbreviations

TNBC: triple-negative breast cancer
OS: overall survival
RFS: recurrence-free survival
DMSF: metastasis-free survival
GO: gene ontology
KEGG: Kyoto Encyclopedia of Genes and Genomes
PPI: Proteome-Protein Interaction
ICIs: immune checkpoint inhibitors
CAR-T: chimeric antigen receptor T-cell therapy
CTRP: The Cancer Therapeutics Response Portal
ROC: receiver operating characteristic
